# Supplementary material for: Dot1l interacts with Zc3h10 to activate Ucp1 and other thermogenic genes
Source: eLife. 2020 Oct 27;9:e59990. doi: 10.7554/eLife.59990 (PMC7661038; doi:10.7554/eLife.59990)
Supplement: Transparent reporting form [file elife-59990-transrepform.docx]

***eLife’s* transparent reporting form**

We encourage authors to provide detailed information *within their submission* to facilitate the interpretation and replication of experiments. Authors can upload supporting documentation to indicate the use of appropriate reporting guidelines for health-related research (see [EQUATOR Network](http://www.equator-network.org/%20)), life science research (see the [BioSharing Information Resource](https://biosharing.org/)), or the [ARRIVE guidelines](http://www.plosbiology.org/article/info:doi/10.1371/journal.pbio.1000412) for reporting work involving animal research. Where applicable, authors should refer to any relevant reporting standards documents in this form.

If you have any questions, please consult our Journal Policies and/or contact us: [editorial@elifesciences.org](mailto:editorial@elifesciences.org).

**Sample-size estimation**

- You should state whether an appropriate sample size was computed when the study was being designed
- You should state the statistical method of sample size computation and any required assumptions
- If no explicit power analysis was used, you should describe how you decided what sample (replicate) size (number) to use

Please outline where this information can be found within the submission (e.g., sections or figure legends), or explain why this information doesn’t apply to your submission:

No explicit power analysis was used. Sample size was decided based on previous experience as N=5-8 for studies using live animals and N=6-8 for biochemical and molecular analyses. Specific sample size and statistical method for each experiment is presented in each Figure legend and the methods section.

**Replicates**

- You should report how often each experiment was performed
- You should include a definition of biological versus technical replication
- The data obtained should be provided and sufficient information should be provided to indicate the number of independent biological and/or technical replicates
- If you encountered any outliers, you should describe how these were handled
- Criteria for exclusion/inclusion of data should be clearly stated
- High-throughput sequence data should be uploaded before submission, with a private link for reviewers provided (these are available from both GEO and ArrayExpress)

Please outline where this information can be found within the submission (e.g., sections or figure legends), or explain why this information doesn’t apply to your submission:

All experiments were repeated at least 2-3 times. Presented data is from representative results from biological replicates, which was defined as biologically distinct samples (e.g. the same genotype of individual animals/cells that were treated/cultured in the same conditions). We did not exclude any mice from in vivo experiments due to statistical reasons. The number of biological replicates in each in vivo study is indicated in the figure legend.

**Statistical reporting**

- Statistical analysis methods should be described and justified
- Raw data should be presented in figures whenever informative to do so (typically when N per group is less than 10)
- For each experiment, you should identify the statistical tests used, exact values of N, definitions of center, methods of multiple test correction, and dispersion and precision measures (e.g., mean, median, SD, SEM, confidence intervals; and, for the major substantive results, a measure of effect size (e.g., Pearson's r, Cohen's d)
- Report exact p-values wherever possible alongside the summary statistics and 95% confidence intervals. These should be reported for all key questions and not only when the p-value is less than 0.05.

Please outline where this information can be found within the submission (e.g., sections or figure legends), or explain why this information doesn’t apply to your submission:

The description of how statistical tests were selected and performed can be found in the Methods section. Statistical comparisons were made using a two-tailed unpaired t-test using GraphPad Prism 8 software (GraphPad Software Inc., La Jolla, CA, USA). Data are expressed as means ± standard errors of the means (SEM). The statistical differences in mean values were assessed by Student's t test.

(For large datasets, or papers with a very large number of statistical tests, you may upload a single table file with tests, Ns, etc., with reference to sections in the manuscript.)

**Group allocation**

- Indicate how samples were allocated into experimental groups (in the case of clinical studies, please specify allocation to treatment method); if randomization was used, please also state if restricted randomization was applied
- Indicate if masking was used during group allocation, data collection and/or data analysis

Please outline where this information can be found within the submission (e.g., sections or figure legends), or explain why this information doesn’t apply to your submission:

In most cases, data analysis was quantitative, so masking was not required. For in vivo experiments (cold exposure, metabolic chamber etc.,), experiments were only performed using mouse IDs, and genotypes were checked at the data analysis stage after the experiments. For in vitro experiments, cells were randomly assigned into groups (control vs. treatment).

**Additional data files (“source data”)**

- We encourage you to upload relevant additional data files, such as numerical data that are represented as a graph in a figure, or as a summary table
- Where provided, these should be in the most useful format, and they can be uploaded as “Source data” files linked to a main figure or table
- Include model definition files including the full list of parameters used
- Include code used for data analysis (e.g., R, MatLab)
- Avoid stating that data files are “available upon request”

Please indicate the figures or tables for which source data files have been provided:

Additional data files are uploaded as Supplementary materials. We are in the process of submitting ATAC-seq and RNA-seq files and depositing in the NCBI database.
